# Supplementary material for: Descriptive Acute Respiratory Distress Syndrome (ARDS) in adults with imported severe Plasmodium falciparum malaria: A 10 year-study in a Portuguese tertiary care hospital
Source: PLoS One. 2020 Jul 9;15(7):e0235437. doi: 10.1371/journal.pone.0235437 (PMC7347120; doi:10.1371/journal.pone.0235437)
Supplement: Attachment — Submitted filename: PONE-D-20-02362.docx [file pone.0235437.s001.docx]

Graca and coworkers examining retrospectively 98 patients with severe *P. falciparum* malaria admitted to their ID-ICU in Portugal between 2008-2018, aimed to identify “predictors” of ARDS development. However, their final conclusion is “ARDS is a hard to predict late complication of severe malaria”. I think that this study is flawed by two main issues: 1) the retrospective enrolment of patients; 2) the limited number of patients. Both contributed to their negative findings.

In their multivariate model only two variables were independently associated with development of ARDS: 1) PaO2/FIO2 < 300 mmHg); 2)SAP < 90 mmHg. However, the first one is now part of the ARDS Berlin definition (previous ALI , now mild ARDS) therefore should not be considered a risk factor for development of ARDS, and the second is, more properly, an indicator of severe malaria.

Given the above mentioned limitations I will suggest to change the purpose of the study from a “predictive” to a “descriptive” of ARDS among patients with severe imported malaria.

Results:

Subheadings

-(Patient demographic and clinical and laboratory characteristics):nationality of patients should be indicated and the area of acquisition of malaria specified. Former Portuguese colonies (either in results and table 1) is ambiguous and the reader is not required to know what they were.

- (Prediction of ARDS development): table 2 shows patients admitted to ID-ICU with (=32) and without ARDS (=66); however , as stated in the text “thirteen of these patients already fulfilled ARDS criteria on admission to the ICU”. The majority of pts developed ARDS during ICU stay (as reported in the literature and also in my experience) and therefore table should be changed accordingly.

It would be interesting to know parasitaemia value at the time of ARDS occurrence in comparison with other criteria (see the article by Marks ME et al. BMC Infect Dis 2013; 13:118).

Also in table 2 there is a discrepancy between “acidosis” (5/57, 0/28) and arterial pH < 7.35 (13/57, 2/29); a pH < 7,35 encompass the definition of acidosis (metabolic acidosis= plasma bicarbonate < 15 mmol /litre or pH < 7.35. WHO Guidelines for the treatment of malaria, 2^nd^ Edition).

-(Management of respiratory failure): this is a descriptive paragraph difficult to follow; I suggest to try to summarize main messages in a table.

Discussion: It would be important to discuss the role of albumin level (high risk factor for both severe malaria and ARDS) (see article Bruneel F et al. Intensive Care Med 2016;42:1588-96)and malaria semi-immunity. Moreover, ARDS is (together with shock and acidosis) one of the factor associated with malaria death but in the present experience none of the patients died with/for ARDS. In a previous article from the same group (Malaria J 2012) regarding 59 pts with severe malaria admitted in the ICU from 2000-2011, 8/9 patients who died with malaria had ARDS. Can you comment on?

Although , your experience is about ARDS in *P. falciparum* malaria, it should be mentioned the role of this complication in *P. vivax* and *P. knowlesi* severe malaria.

Minor:

“Immunochromatogenic” change to ” immunochromatographic”

Reference 12 refers to India, actually to be considered an endemic country for malaria.

Table 1: seven patients were immunocompromised: 3 had HIV infection; I do not agree that HIV “per se” should be considered immunocompromission.
